# Peripartum cardiomyopathy: ten year experience at a tertiary care hospital in Pakistan

**DOI:** 10.1186/1756-0500-6-495

**Published:** 2013-12-01

**Authors:** Abid Hussain Laghari, Aamir Hameed Khan, Khawar Abbas Kazmi

**Affiliations:** 1 Department of Medicine, Aga Khan University Hospital, Karachi, Pakistan

**Keywords:** Peripartum cardiomyopathy, Left ventricular systolic dysfunction, Right ventricular dysfunction

## Abstract

**Background:**

There is very little literature regarding peripartum cardiomyopathy from the Asian countries. We conducted this study to determine demographic details, clinical presentations, complications and recovery of left ventricular (LV) systolic function in peripartum cardiomyopathy (PPCMP) patients of Pakistani origin.

**Method:**

A ten year retrospective case series of PPCMP was conducted at the Aga Khan University Hospital. Patients were also followed up for six months after presentation, with special regard to improvement in the LV function.

**Results:**

Total 45 patients were included, 25 (55.5%) primigravida and 8 (17.7%) gravida 2 and the remaining 12 (26.6%) were multigravida. Fourteen patients (31.1%) presented during pregnancy and 31 (68.8%) after delivery. All patients presented with CHF and three (6.6%) were complicated with ventricular tachycardia (VT) at presentation. LV systolic dysfunction was present in 39 (86.66%) patients and RV dysfunction in 15 (33.3%) patients. Two patients had LV clot and thromboembolic stroke occurred in another 4 patients. All patients received standard treatment except three patients who had asthma and could not be given beta blockers. Echocardiogram was repeated after 6 month and in 32 (71.1%) patients LV functions recovered to normal. RV function improved in all except 2 (4.4%) patients. All patients were discharged in stable condition.

**Conclusion:**

Significant numbers of PPCMP patients, who had severe LV dysfunction at presentation recovered their LV functions at six month follow up.

## Background

Peripartum cardiomyopathy (PPCMP) is a rare cause of heart failure that affects women in the last month of pregnancy or within 5 months of delivery [1]. PPCMP was initially described in 1849 [2]. Other terms used for this condition are toxic postpartum heart failure, Zaria syndrome, Meadows’ syndrome and postpartum myocardiosis. The incidence of PPCMP varies extensively from 1:15,000 to 1:100 deliveries across geographic regions of world [3]. The exact cause of the condition is not known, with possible etiologies including genetic, inflammatory, autoimmune and hormonal factors [4-6]. Management is similar to that employed for other types of heart failure with left ventricular systolic dysfunction with care however, to ensure the safety of the mother and the unborn or breastfed baby. Cardiac transplantation is often the only acceptable treatment for patients in whom conventional therapy is not successful. A number of factors are associated with increased risk, including age greater than 30 years [7], multiparity, African descent, pregnancy with multiple fetuses [8], history of preeclampsia, eclampsia, or postpartum hypertension, maternal cocaine abuse, long-term (>4 weeks) oral tocolytic therapy with beta adrenergic agonists like terbutaline [9]. The echocardiogram commonly reveals a global reduction in LV contractility and enlargement without hypertrophy [10]. Our purpose was to study the clinical profile of all patients that presented to our center with this diagnosis.

## Methods

This was a descriptive study (case series) done at the Aga Khan University Hospital (AKUH) by reviewing the patients’ medical files from 1st June 2001 to 30th May 2011. The study was conducted after the ethical approval was received from the University ethics committee. Files of the patients diagnosed with peripartum cardiomyopathy were reviewed for age of the patient, gravid status, number of fetuses, presenting features, clinical course and cardiac complications during the index hospital stay. Echocardiographic parameters including LV ejection fraction, RV function and mitral regurgitation (MR) were noted at presentation and after six months to see the course of functional improvement in the systolic function.

### Diagnostic criteria for PPCM

(1) Development of heart failure in the last month of pregnancy or within 5 months of delivery; (2) absence of another identifiable cause for the heart failure; (3) absence of recognizable heart disease prior to the last month of pregnancy; (4) left ventricular ejection fraction (LVEF) less than 45% percent.

### Recovery of LV function

Was defined as echocardiographic documentation of left ventricular ejection fraction greater than or equal to 50%.

### Standard treatment

All patients in the study received standard treatment including loop diuretics, angiotensin converting enzyme inhibitor (ACE inhibitor) / ARBs and beta-blockers, except the few patients who had contraindication to specific medicine.

## Results

Forty five patients of PPCMP were found through the diagnosis coding system and their files were reviewed. Patient age range was between 19 and 40 years (mean age 27.4 ± 6.05). Majority of the (Table 1) patients 25 (55.5%) were primigravida and 8 (17.7%) patients were gravida 2, remaining 12 (26.6%) patients were multigravida. Three patients had twin fetuses. Fourteen patients (31.1%) presented during pregnancy and the remaining 31 (68.8%) patients presented after delivery. Presentation after delivery ranged from two days to two months. All patients presented with symptoms and signs of heart failure, four (8.8%) patients additionally had preeclampsia and one had eclampsia. Three (6.6%) were complicated with ventricular tachycardia at presentation. Majority of the patients (86.66%) had severe left ventricular systolic dysfunction (EF <30%) and 6 (13.3%) had moderate left ventricular systolic dysfunction (EF <45% and >30%). Similarly 17 (37.7%) patients had severe mitral regurgitation (MR), 7 (15.5%) had moderate MR and 14 (31.1%) patients had mild MR. RV dysfunction was noted in 15 (33.3%) patients. Two patients had LV clot and hospital course was complicated by thromboembolic stroke in another four patients. All patients received standard treatment except three patients who had asthma and could not be administered beta blockers. Echocardiograms were repeated after six month and the majority (71.1%) had recovered the LV functions (EF >50%). Thirteen (28.8%) patients did not recover LV function and had severe LV dysfunction (Table 2) (EF <30%) at six months. In 13 patients who did not recover their LV function; four had severe MR. In the group of patient in whom LV function did not improve, majority (76.92%) had age over 30 years, five (38.14) patients were gravida 2 and three (23.07%) patients were multigravida. The patients who improved LV function at six months; there also was a significant improvement in the MR. Right ventricular function improved in the majority of patients, only two patients (4.4%) had residual RV dysfunction at six months. In our cohort all the patients were discharged from hospital in a stable condition.

**Table 1:** Patient’s demographics at presentation

Number of cases (n)	45
Age range years	19 to 40
Mean age years	27.4 ± 6.05
Primigravida	25 (55.5%)
Multigravida	20 (44.4%)
Twin pregnancy	03 (6.6%)
Preeclampsia	04 (8.8%)
Eclapmsia	01 (2.2%)
Presentation during pregnancy	14 (31.1%)
Presentation after delivery	31 (68.8%)
Presentation (Average days after delivery)	02 - 60
Severe LV systolic dysfunction	39 (86.6%)
Moderate LV systolic dysfunction	06 (13.3%)
Severe MR	17 (37.7%)
Moderate MR	07 (15.5%)
Mild MR	14 (31.1%)

**Table 2:** Presentation, complication, LV and RV improvement

CHF	45 (100%)
Arrhythmias (VT)	03 (6.6%)
Stroke (Ischemic)	04 (8.8%)
LV clot	02 (4.4%)
LV EF Improved (EF >50%)	32 (71.1%)
RV Function improved	43 (95.5%)
RV Dysfunction at 6 months	02 (4.4%)

## Discussion

The incidence of peripartum cardiomyopathy (PPCMP) varies extensively across geographic regions of world from 1:15,000 to 1:100 deliveries [3]. There is very little literature from Asian countries. In a study from south India the incidence of PPCMP has been reported at 1 case per 1374 live births [11]. The reported high incidence of PPCMP in Nigeria (one case per 102 deliveries) [12] may have an association to the local Hausa custom of eating kanwa, a dry lake salt for 40 days after delivery [3]. The exact cause of PPCMP is still unknown and may be multifactorial. Clustering of PPCMP in families has been also published in case reports [13]. The hemodynamic stress of gestational hypertension may contribute to the development of heart failure [5]. However, in our study only five (11.1%) patients had gestational hypertension. Since the etiology of PPCMP is still unclear, a number of factors have been associated with increased risk, including age greater than 30 years, multiparity, African descent [14], pregnancy with multiple fetuses [8], a history of preeclampsia, eclampsia, postpartum hypertension, maternal cocaine abuse, long-term (>4 weeks) oral tocolytic therapy with beta adrenergic agonists such as terbutaline [9]. In our study 13 (28.8%) patients were more than 30 years age, 6.6% of patients had twin pregnancy and four patients had preeclampsia. One patient was diagnosed with eclampsia. Three patients had asthma, who received salbutamol inhalers (Beta agonist). Twenty (44.4%) patients were multigravida. At our center patients most commonly presented with symptoms of heart failure. In our study four patients were complicated by cerebral thromboembolic events during the clinical course. The echocardiogram usually reveals a global reduction in contractility and LV enlargement without hypertrophy [10]. The majority of our patients had global left ventricular dysfunction and 1/3rd had right ventricular dysfunction as well. Other echocardiographic findings include, regional heterogeneities of systolic wall thickening, left atrial enlargement, mitral and tricuspid regurgitation [10]. The majority of our patients had associated MR and most improved with improvement in the LV functions. Atrial fibrillation occurs occasionally in patients with PPCMP [12]. Sustained ventricular tachycardia has rarely been reported with PPCMP. Three of our patients (6.6%) had ventricular tachycardia (VT) at presentation. Patients with PPCMP are at high risk for thrombus formation and thromboembolism due to the hypercoagulable state of pregnancy and the stasis of blood secondary to severe LV dysfunction [15]. Four of our patients had embolic ischemic events and two patients had LV apical thrombus. Limited data are available to guide the timing and mode of delivery in PPCM. According to the 2010 European society of cardiology working group statement, early delivery is not required if the maternal and fetal conditions are stable [16]. A number of studies have evaluated the outcome of women with PPCMP [17]. The largest series of 123 cases of PPCMP showed a cardiac transplantation rate of 4% and a mortality rate of approximately 10% at a mean follow-up of about 2 years [17]. Recovery to an LVEF above 50 percent occurred in 54% of patients, and the degree of recovery was greatest in those with a baseline LV ejection fraction >30% [17,18]. In our study 71.1% of PPCMP patients recovered left ventricular ejection fraction and the mitral regurgitation also improved in this group. Baseline LVEF has limited sensitivity for prediction of improvement in individual patients [19]. Most of our patients (86.6%) had severe LV systolic dysfunction at presentation.

## Conclusion

In our study majority of the patients had severe LV dysfunction at time of presentation and a significant number had recovery in their LV function at 6 months. Other than more observed ventricular arrhythmias at presentation, our study results are congruent with the international literature.

## Abbreviations

PPCMP: Peripartum cardiomyopathy; LV: Left ventricle; RV: Right ventricle; EF: Ejection fraction; VT: Ventricular tachycardia; CHF: Congestive heart failure.

## Competing interests

The authors declare that they have no competing interests.

## Authors’ contributions

AHL was responsible for concept, drafting the protocol, getting approvals, entering data and doing the analysis. KAK was responsible in revising and proof reading of the study. AHK was responsible for the concept, revising and proof reading of the study. All authors read and approved the final manuscript.

## References

[B1] Hasan JA Ramejo BB Qureshi A Kamran A Peripartum cardiomyopathy characteristics and outcome in a tertiary care hospital J Pak Med Assoc 2010 64 5 377 380 20527612

[B2] Richie C Clinical contribution to the pathology, diagnosis and treatment of certain chronic diseases of the heart Edinb Med Surg J 1849 2 333 PMC5792849 30330805

[B3] Sliwa K Damasceno A Mayosi BM Epidemiology and etiology of cardiomyopathy in Africa Circulation 2005 112 3577 3583 10.1161/CIRCULATIONAHA.105.542894 16330699

[B4] Sliwa K Fett J Elkayam U Peripartum cardiomyopathy Lancet 2006 368 687 693 10.1016/S0140-6736(06)69253-2 16920474

[B5] Murali S Baldisseri MR Peripartum cardiomyopathy Crit Care Med 2005 33 S340 S346 10.1097/01.CCM.0000183500.47273.8E 16215357

[B6] Hilfiker-Kleiner D Kaminski K Podewski E Bonda T Schaefer A Sliwa K Forster O Quint A Landmesser U Doerries C Luchtefeld M Poli V Schneider MD Balligand JL Desjardins F Ansari A Struman I Nguyen NQ Zschemisch NH Klein G Heusch G Schulz R Hilfiker A Drexler H A cathepsin D-cleaved 16 kDa form of prolactin mediates postpartum cardiomyopathy Cell 2007 128 589 600 10.1016/j.cell.2006.12.036 17289576

[B7] Demakis JG Rahimtoola SH Sutton GC Meadows WR Szanto PB Tobin JR Gunnar RM Natural course of peripartum cardiomyopathy Circulation 1971 44 1053 1061 10.1161/01.CIR.44.6.1053 4256828

[B8] Homans DC Peripartum cardiomyopathy N Engl J Med 1985 312 1432 1437 10.1056/NEJM198505303122206 3887170

[B9] Lampert MB Hibbard J Weinert L Briller J Lindheimer M Lang RM Peripartum heart failure associated with prolonged tocolytic therapy Am J Obstet Gynecol 1993 168 493 495 10.1016/0002-9378(93)90479-3 8438916

[B10] Lampert MB Lang RM Peripartum cardiomyopathy Am Heart J 1995 130 860 870 10.1016/0002-8703(95)90089-6 7572598

[B11] Vinay P Sirish S Ashwini K Afrin S Incidence and outcome of peripartum cardiomyopathy from a tertiary hospital in South India TROPICAL DOCTOR 2009 39 168 169 10.1258/td.2008.080353 19535757

[B12] Isezuo SA Abubakar SA Epidemiologic profile of peripartum cardiomyopathy in a tertiary care hospital Ethn Dis 2007 17 2 228 233 17682350

[B13] Pearl W Familial occurrence of peripartum cardiomyopathy Am Heart J 1995 129 421 422 10.1016/0002-8703(95)90032-2 7832124

[B14] Veille JC Peripartum cardiomyopathies: a review Am J Obstet Gynecol 1984 148 805 818 10.1016/0002-9378(84)90572-6 6367478

[B15] Walsh JJ Burch GE Black WC Ferrans VJ Hibbs RG Idiopathic myocardiopathy of the puerperium (Postpartal Heart Disease) Circulation 1965 32 19 31 10.1161/01.CIR.32.1.19 14314486

[B16] Sliwa K Hilfiker-Kleiner D Petrie MC Mebazaa A Pieske B Buchmann E Regitz-Zagrosek V Schaufelberger M Tavazzi L van Veldhuisen DJ Watkins H Shah AJ Seferovic PM Elkayam U Pankuweit S Papp Z Mouquet F McMurray JJ Heart Failure Association of the European Society of Cardiology Working Group on PeripartumCardiomyopathy: Current state of knowledge on aetiology, diagnosis, management, and therapy of peripartum cardiomyopathy: a position statement from the Heart Failure Association of the European Society of Cardiology Working Group on peripartum cardiomyopathy Eur J Heart Fail 2010 12 767 778 10.1093/eurjhf/hfq120 20675664

[B17] Amos AM Jaber WA Russell SD Improved outcomes in peripartum cardiomyopathy with contemporary Am Heart J 2006 152 509 513 10.1016/j.ahj.2006.02.008 16923422

[B18] Elkayam U Akhter MW Singh H Khan S Bitar F Hameed A Shotan A Pregnancy associated cardiomyopathy: clinical characteristics and a comparison between early and late presentation Circulation 2005 111 2050 2055 10.1161/01.CIR.0000162478.36652.7E 15851613

[B19] Goland S Bitar F Modi K Safirstein J Ro A Mirocha J Khatri N Elkayam U Evaluation of the clinical relevance of baseline left ventricular ejection fraction as a predictor of recovery or persistence of severe dysfunction in women in the United States with peripartum cardiomyopathy J Card Fail 2011 17 426 430 10.1016/j.cardfail.2011.01.007 21549301

